# Analysis of salivary level *Lactobacillus* spp. and associated factors as determinants of dental caries amongst primary school children in Harar town, eastern Ethiopia

**DOI:** 10.1186/s12887-020-1921-9

**Published:** 2020-01-16

**Authors:** Dawit Ademe, Desalegn Admassu, Senthilkumar Balakrishnan

**Affiliations:** 1 Department of Medical Laboratory Sciences, College of Health and Medical Sciences, Haramaya University, P.O. Box: 235, Harar, Ethiopia; 2 Department of Medical Microbiology, College of Health and Medical Sciences, Haramaya University, P.O. Box: 235, Harar, Ethiopia

**Keywords:** Dental caries, Associated factors, DMFT, *Lactobacillus*, Schoolchildren

## Abstract

**Background:**

*Lactobacillus* spp. play a major role in the development of dental caries. Although effective methods are known for the prevention and management of dental caries, its prevalence of dental caries is increasing amongst school-age children in Ethiopia. Hence, this study is aimed to determine the association of salivary *Lactobacillus* spp. level and oral health factors as determinants of dental caries amongst primary school children in Harar town, Eastern Ethiopia.

**Methods:**

A cross-sectional study was conducted amongst primary school children (*n* = 407) using a questionnaire survey, clinical DMFT (decayed, missing, filled tooth number) examination and enumeration of salivary level *Lactobacillus* spp. Data were analyzed by SPSS (Statistical Package for Social Science software- version 25.0). The odds ratio was used to determine the predictors of the outcome. The data were expressed as mean ± SD. Statistical significance was defined as a *p*-value of less than 0.05.

**Results:**

The prevalence of dental caries was found to be 36.9% amongst primary school children. The mean DMFT value of the participants was 0.95 ± 1.57. The dental decay component was the primary observation that accounted for 84.6% of the DMFT. Almost, 67% of the saliva culture revealed a significant amount of *Lactobacillus* spp. count. The mean bacterial count was found to be 14.92 × 10^5^ ± 22.92 × 10^5^. Among various parameters analysed for association with dental caries, a number of them show positive associtation, incluing reduction in academic scores in the prevous academic year (*p* = 0.034), grade levels of school children 1–4 (*p* = 0.041), sweet food consumption habit (*p* = 0.003), absence of daily teeth cleaning habit (*p* = 0.002), absence of toothpaste use (*p* = 0.001), dental ache history (*p* = < 0.001), significant microbial load of salivary *Lactobacillus* spp. (*p* = < 0.001), acidic (*p* = 0.028) and basic salivary pH (*p* = 0.025).

**Conclusion:**

A significant salivary *Lactobacillus* count associated with lower grade level, sweet diet, and poor oral hygiene were found to be the determinant factors for dental caries. Thus, dissemination of oral health information is obligatory to prevent dental caries amongst primary school children in the study area.

## Background

Dental caries is a complex and dynamic process where a multitude of factors initiates and influence the progression of the disease [1]. It has been known throughout history but started to become a significant health problem in industrialized countries in the latter part of the nineteenth century, when new technologies allowed the production of large amounts of refined sugars. Globally prevalence indicates about 60–90% of children and nearly 100% of the adult world population is suffering from dental caries [2, 3]. While WHO reports that untreated caries accounts for 44% dental problems in 2010 [4], lack of oral hygiene practices is common problem in many developing and developed countries [5, 6].

Tooth decay or cavity is a breakdown of teeth due to bacterial activity. It is generally caused by association of multiple microorganisms and improper oral hyegiem practices [7]. *Streptococcus mutans* and *Lactobacillus* spp. are the most common pathogens isolated from human dental plaque and are considered as the major etiologic agents of caries. However, there is a central role for *S. mutans* in the initiation and *Lactobacillus* spp. in the major progression of dental caries [8]. In addition, risk factors such as host susceptibility [9], age [10], dietary habits [11], socioeconomic and oral hygiene status [12] have been associated with increased incidence of dental caries [3, 13, 14] in human population.

Dental caries is found to be one of the five health problems of school children followed by respiratory diseases, malnutrition, intestinal parasites, skin, eye and ear diseases [2]. Children spend most of the active hours in schools, therefore, hygeinic leassons at schools are better strategies to educate children about oral health. Oral health appraisal in school might consists of determining the total health status of the child through health histories, teacher and nurse observations, medical screening tests, dental examination, and and basic education on oral hygiene [1, 15].

However, the lack of locally accessible data to diagnose and treat caries is the central issue in controlling dental caries. Although dental caries has been affecting the majority of the Ethiopian children, much is unknown about the extents and factors that influencing the occurrence. Therefore, this study was intended to determine the prevalence, association of salivary level *Lactobacillus* spp. and relevant factors as derteminant for dental caries amongst primary school children. This is the first report that analyse the prevalence and factors associated for dental caries in this region, thus it could serve as a baseline for the regional health bureau, non-governmental organizations, researchers and policy makers for considering the interventions strategies to prevent dental caries in Ethiopia.

## Methods

### Study period and locality

A cross-sectional study was conducted from February 7 to March 14, 2018, in primary schools, Harar town, Eastern Ethiopia. Harar is a capital town of Harari Regional State and it is located 515 km away from the capital city, Addis Ababa.

### Population

A total of 5 government primary schools was selected and all children who were present in the school during data collection were included in the study.

### Sample size determination

The sample size for the prevalence of dental caries was calculated by using a single population proportion formula. The sample size for associated factors was determined using Epi info (version 7) at a confidence interval (1-α) =95% and power (1-β) of 80%. Finally, a 10% non-response rate was added to the calculated sample size. Therefore, the largest sample size, i.e. 422 was used for the study.

### Sampling

A total of 422 children was included from 5 schools, out of the total 20 government primary schools in Harar town. The selection of school children for the study was detrmined as follows. First, the five primary schools were selected based on the lottery method. Secondly, ten sections from both the first cycle and second cycle level were selected by simple random sampling. Thirdly, students were distributed proportionally amongst each section. Finally, participants were selected based on the list from the class teacher’s who is in - charge for the class using systematic random sampling. In the case of absenteeism and overlap, the next number was included in the study.

### Data collection

A structured questionnaire was used to collect information about the socio-demographic characteristics and determinant factors relevant for dental caries amongst primary school children. Data were collected by face-to-face interview with the help of the children’s parents or guardians. Dietary data were collected using the Ethiopian demographic and health survey questionnaires as a baseline to estimate the habitual intake of foods and nutrients [16]. Interviewers collected information about the foods and drinks consumed during the preceding day by probing questions and cues that help the recollection of participant’s memory. Two new versions were developed for this survey: version C2 for children aged 3–11 years designed for completion by a parent/guardian with help from the child, and version C3 for young people aged 12–17 years for completion by the young person with help from their parent/guardian. Both questionnaires list 15 foods or drinks with a measure defined for each item. Participants were asked to estimate the frequency and amount of each food or drink consumed per day.

### Clinical examination

A trained dental therapist (nurse) and a dentist (doctor) with assistant data recorder performed the clinical examination in the classrooms. A dental visual examination was performed on each child by one examiner using a light-emitting diode (LED), dental mirror, and explorer. The diagnostic criteria for caries were followed according to the WHO recommendations [17]. The DMFT (decayed, missing, and filled tooth number) scores were recorded and dental decay is measured using the count of the number of teeth or surfaces in a child’s that are decayed, missing or filled as a result of caries. A tooth was recorded as decayed when a lesion had an unmistakable cavity, undetermined enamel or detectably softened wall or floor. A tooth was recorded as missing when it was extracted due to caries. A tooth was recorded as filled when it was permanently filled without caries. The examiner was calibrated with an experienced dentist (doctor) in the same setting before the study. The intra-examiner agreement was assessed by re-examining a 10% random sample of the children on the same day. The dental assistant without the knowledge of the examiner performed the selection of children for duplicate examination.

### Specimen collection, culture, and identification

Unstimulated whole saliva samples (2 mL) were collected from children [18] by a medical laboratory technologist. Children were requested to void saliva into a wide-mouthed disposable cup 2 h after their last meal in a mid-morning. The cup was then uniquely coded, kept in ice box, and transported for microbiological assay within 24 h.

Saliva (0.1 mL) was diluted with normal saline (0.9 mL) and tenfold serial dilutions of saliva were made up to 10^− 6^. The diluted sample (100 μL) was spread [19] evenly onto de Man, Rogosa and Sharpe agar (MRS agar) (HI Media Laboratories Pvt. Ltd., India) to obtain direct counts of *Lactobacilli*. Plates were incubated anaerobically using the anaerobic candle jar system at 37 °C. After 48 h of incubation, plates were observed for the presence of *Lactobacillus* like colonies. Additionally, Gram staining or catalase test [19–21] was performed.

Colony counting was performed using a colony counter and the number of colony-forming units (CFU) was multiplied by the number of times the original milliliter of sample diluted (the dilution factor of the plate counted) and expressed as CFU/mL [19] of saliva. Besides, the determination of the salivary pH for each sample was performed, using a special pH-test paper (Simplex Health pH test strips for urine and saliva: manufacturer part number sh003, United Kingdom). The threshold defined by the manufacturer made an interpretation.

### Data quality control

A trained dental therapist performed clinical examination based on the WHO oral health assessment. Similarly, a trained medical laboratory technologist performed the laboratory diagnosis. A pretested and structured questionnaire used to collect data. The questionnaire was developed in the English language and translated to appropriate Ethiopian local languages (Amharic and Afan Oromo). Double entry was performed before data analysis for validation. Study participants were required to refrain from eating and drinking for a minimum of 2 h after arrival at school prior to sample collection.

Culture examinations were undertaken after checking growth-supporting characteristics, physical characteristics, gel strength and batch contamination of the media used. Prepared media quality was monitored by incubating 3–5% of the batch at 35–37 °C for overnight. The reference strain of *Lactobacillus casei* (ATCC 393) and *Lactobacillus fermentum* (ATCC 9338) obtained from Ethiopia Public Health Institution (EPHI), Addis Ababa were used for quality control of culture.

### Data analysis

Data were checked for completeness, cleared and then entered and validated by Epi data, software version 3.1 and exported to the Statistical Package for Social Science (SPSS) software version 25.0 for analysis. Odds ratios (OR) and their 95% confidence intervals (CI) were estimated using bivariate and multivariate logistic regression analysis to identify possible explanatory variables on the occurrence of dental caries. The data were expressed as mean ± SD. The *p*-value < 0.05 was considered as statistically significant.

## Results

### Socio-demographic characteristics of primary school children

A total of 407 children was included in the study giving a response rate of 98%. Of these, 214 (52.6%) were boys and 193 (47.4%) were girls. About 156 (38.3%) and 251 (61.7%) of the students were from the 1st cycle (grades 1–4) and 2nd cycle (grades 5–8), respectively. Most of the participants were Muslim (87.7%). The Oromo (64.1%), Harari (17.9%) and Amhara (6.9%) were amongst the study population. Nearly two-thirds (61.9%) of the participants were living in an urban area (Table 1).

**Table 1:** Socio-demographic characteristic of study participants amongst primary school children in Harar, Eastern Ethiopia, 2018

Variables	Number (%)
Age in years	
6–11	107 (26.3)
12–15	300 (73.7)
Sex	
Boys	214 (52.6)
Girls	193 (47.4)
Grade	
1–4	156 (38.3)
5–8	251 (61.7)
Residence	
Urban	252 (61.9)
Rural	155 (38.1)
Ethnicity	
Oromo	261 (64.1)
Amhara	28 (6.9)
Harari	73 (17.9)
Gurage	17 (4.2)
Somali	8 (2)
Tigray	2 (0.5)
Other	18 (4.4)
Religion	
Orthodox	37 (9.1)
Muslim	357 (87.7)
Protestant	7 (1.7)
Catholic	4 (1)
Other	2 (0.5)
Average academic score (%)	
< 50	24 (5.9)
> 50	383 (94.1)

### Food consumption pattern, dietary habits and practices related to oral hygiene

In the case of children’s dietary habits, it was found that 243 (59.7%) had bread with tea for breakfast, 310 (76.2%) drank up to 1500 mL of soft drinks per week and 358 (88%) of them had taken various quantities of sweet foods on a daily basis. The mean salivary pH was found to be 6.5 ± 1.1. About 48.6% of the children showed acidic salivary pH.

Of the total children, 36.9% did not clean their teeth at least once a day. However, the majority of second cycle students (71%) had teeth cleaning habits of which 75.7% used a brush with toothpaste. About 48% of the children with dental caries neither had a tooth cleaning habit with a wooden stick nor with a toothbrush and 45.3% had no mouth rinsing habits after each meal (Table 2).

**Table 2:** Factors associated with dental caries amongst primary school children in Harar, Eastern Ethiopia, 2018

Variables	Number (%)
Bread as a major staple food	
Yes	243 (59.7)
No	164 (40.3)
Pasta as major staple food	
Yes	211 (51.8)
No	196 (48.2)
Consumption of sugared tea (Daily)	
Yes	367 (90.2)
No	40 (9.8)
Consumption of soft drinks (Daily)	
Yes	310 (76.2)
No	97 (23.8)
Consumption of sweet foods (Daily)	
Yes	358 (88.0)
No	49 (12.0)
Daily cleaning teeth	
Yes	257 (63.1)
No	150 (36.9)
Way of cleaning teeth	
Toothbrush with paste	126 (49.0)
Tooth stick	115 (44.7)
Toothbrush and tooth stick	16 (6.2)
Rinse with water (After each meal)	
Yes	354 (87.0)
No	53 (13.0)
Dental ache history	
Yes	148 (36.4)
No	259 (63.6)
Treatment seeking places for dental ache	
Health institution	66 (16.2)
Pharmacy	17 (4.2)
Traditional healer	11 (2.7)
Nowhere	313 (76.9)
Salivary pH	
Acidic (< 6.5)	198 (48.6)
Neutral (6.75–7.25)	127 (31.2)
Basic (> 7.25)	82 (20.1)

### Prevalence of dental caries amongst primary school children

**Fig. 1:**
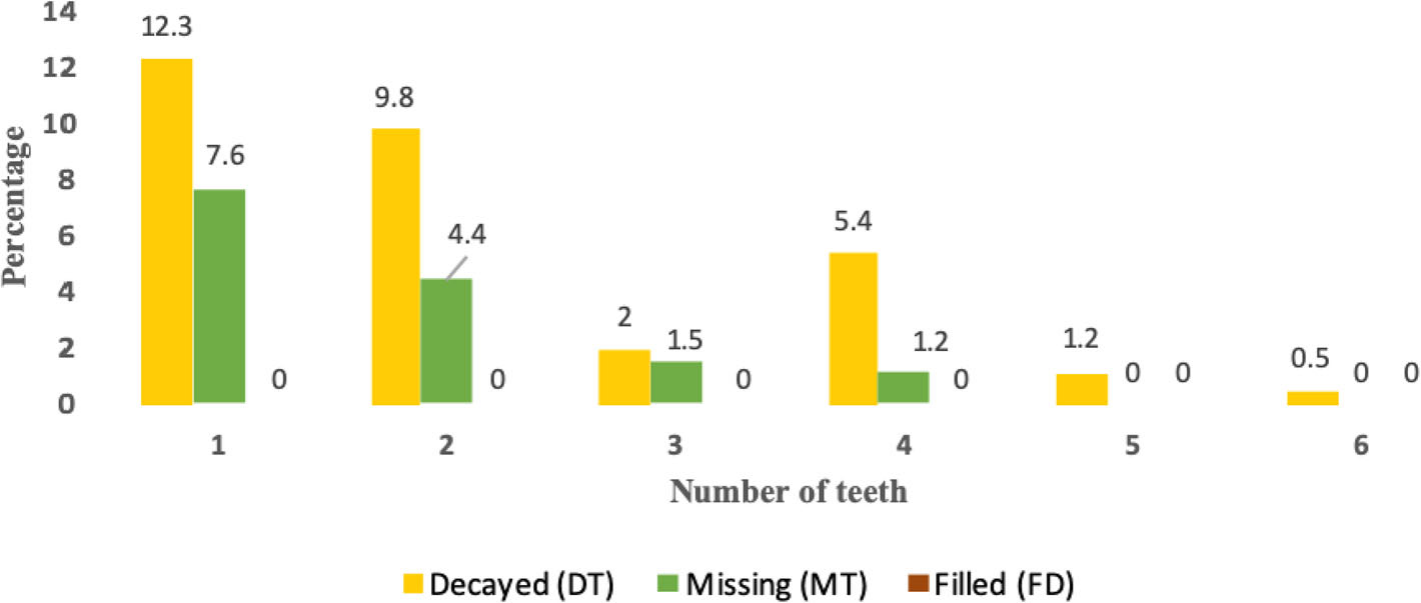
Distribution of decayed, missing and filled teeth in primary school children in Harar, Eastern Ethiopia, 2018

About 150 (36.9%) of the respondents had at least one carious tooth during examination (Fig. 1). Students from a rural setting had a nearly similar prevalence of caries with urban (38.1% versus 36.3%). The occurrence of dental caries was also similar between female (36.8%) and male respondents (36.9%). About 42.7% of the first cycle and 33.7% of second cycle students had caries. Amongst children with caries, 54.5% of them had previous year average academic scores less than 50 % (Table 3).

**Table 3:** Determinant factors of dental caries amongst primary school children in Harar, Eastern Ethiopia, 2018

Variables	Dental caries		COR (95% CI)	AOR (95% CI)	*P*-value
	Yes	No			
	No. (%)	No. (%)			
Grade					
1–4	61(42.7)	82(57.3)	2.07 (1.37–3.14)	1.73 (1.02–2.92)	0.041
5–8	89(33.7)	175(66.3)	1	1	
Previous year average academic score (%)					
Less than 50	6(54.5)	5(45.5)	7.31 (2.66–20.0)	3.68 (1.1–12.29)	0.034
50 and greater	131(34.2)	252(65.8)	1	1	
Consumption of sweet foods					
Yes	140(39.2)	217(60.8)	2.5 (1.21–5.17)	4.08 (1.62–10.2)	0.003
No	10(20.4)	39(79.6)	1	1	
Cleaning teeth					
No	72(48)	78(52)	3.57 (2.34–5.45)	2.23 (1.32–3.78)	0.002
Yes	78(30.4)	179(69.6)	1	1	
Way of cleaning teeth					
Toothbrush without paste	97(39.4)	149(60.6)	0.26 (0.16–0.42)	3.05 (1.69–5.49)	0.001
Toothbrush with paste	52(32.9)	106(67.1)	1	1	
Dental ache history					
Yes	79(53.4)	69(46.6)	3.03 (1.99–4.62)	2.95 (1.78–4.89)	< 0.001
No	71(27.4)	188(72.6)	1	1	
Salivary *Lactobacillus* count					
Significant	125(45.8)	148(54.2)	3.68 (2.24–6.04)	4.01 (2.24–7.18)	< 0.001
Insignificant	25(18.4)	109(81.3)	1	1	
Salivary pH					
Acidic (< 6.5)	58(42.9)	113(57.1)	2.87 (1.75–4.72)	1.93 (1.07–3.48)	0.028
Basic (> 7.25)	27(32.9)	55(67.1)	0.83 (0.41–1.69)	0.38 (0.17–0.88)	0.025
Neutral (6.75–7.25)	38(29.9)	89(70.1)	1	1	

*COR* crude odds ratio, *AOR* adjusted odds ratio, *CI* confidence interval P-value < 0.05

### DMFT score of the primary school children

The mean DMFT value of the participants was 0.95 ± 1.57. The decayed teeth (DT) component had a mean value of 0.69 ± 1.26, contributing 84.6% to the DMFT value. The missing teeth (MT) contributed the rest, 15.4% (Table 4). Females had a DMFT score of 0.94 ± 1.59 and males had 0.95 ± 1.55. Students from the Urban setting had a mean DMFT of 0.92 ± 1.52 compared to rural students with a mean DMFT of 0.99 ± 1.66. Second cycle students had less decayed and missing components compared to the first cycle students. Boys and girls had almost similar DMFT scores.

**Table 4:** Components of DMFT score of primary school children in Harar, Eastern Ethiopia 2018

Variable	Component of DMFT Score	
	$\overline{\mathrm{X}}$ ± SD	Percentage
Decayed (DT)	0.69 (±1.26)	84.6
Missing (MT)	0.26 (±0.72)	15.4
Filled (FT)	0	0
DMFT	0.95 (±1.57)	100

### Enumeration and identification of *Lactobacillus* spp. from saliva

Significant *Lactobacillus* spp. count (≥ 10^5^ CFU) was identified in 67.1% of the saliva samples. The mean bacterial count was 14.92 × 10^5^ ± 22.92 × 10^5^. A statistically significant association was found between caries and the salivary level of *Lactobacillus* spp. (AOR = 4.01; 95% CI: 2.24–7.18; *p* = < 0.001)] (Table 3).

### Determinants of dental caries

In the bivariate logistic regression analysis; age (COR = 1.49; 95% CI: 0.95–2.35; *p* = 0.07), girls (COR = 1.00, 95% CI: 0.67–1.50 *p* = 0.078), urban residency (COR = 0.92; 95% CI: 0.60–1.39; *p* = 0.075) were not statistically associated with dental caries. On the other hand, grade level 1–4 (COR = 2.07; 95% CI: 1.37–3.14; *p* = 0.001) and previous year average academic score less than 50% (COR = 7.31; 95% CI: 2.66–20.0; *p* = < 0.001) were found significant associations with dental caries.

In the rest bivariate logistic regression analysis; consumption of sweet foods (COR = 2.5; 95% CI: 1.21–5.17; *p* = 0.002), absence of daily tooth cleaning habit (COR = 3.57; 95% CI: 2.34–5.45; *p* = 0.002), absence of toothpaste use (COR = 0.26; 95% CI: 0.16–0.42; *p* = 0.011), dental ache history (COR = 3.03; 95% CI: 1.99–4.62; *p* = 0.031), significant salivary *Lactobacillus* spp. count (COR = 3.68; 95% CI: 2.24–6.04; *p* = < 0.001), and acidic salivary pH (COR = 2.87; 95% CI: 1.75–4.72; *p* = 0.001) of the children had significant association with dental caries (Table 3).

### Factors associated with dental caries amongst primary school children

Variables with *p* < 0.25 from the bivariate analysis were entered into multivariate analysis to control possible confounders and to identify determinant factors for dental caries. Variables with *p* < 0.05 were considered statistically significant. On multivariate analysis grade levels of children, previous year average score, tooth cleaning habit, use of a toothbrush with paste, presence of toothache, salivary *Lactobacillus* spp. level, saliva with acidic pH, and sweet food consumption habit remained significant predictors of dental caries. Besides, basic salivary pH was significantly associated with the model (Table 3).

There was a statistically significant difference in the development of dental caries based on the children’s grade level. Children at grade level 1–4 were about 2 times more likely to develop dental caries than grade level 5–8 (AOR = 1.73; 95% CI = 1.02–2.92). Primary school children who had dental caries were four times more likely to score < 50% average compared to children without dental caries (AOR = 3.68; 95% CI = 1.1–12.29). Children who did not clean their teeth were 2 times more likely to have caries than those who cleaned (AOR = 2.23; 95% CI = 1.32–3.78). Moreover, children who did not use a toothbrush with paste for cleaning their teeth were 3 times greater risk to have dental caries than those who did not (AOR = 3.05; 95% CI = 1.69–5.49). The odds of having dental caries were significantly higher amongst children who suffered from toothache than those children who had not (AOR = 2.95; 95% CI = 1.78–4.89) (Table 3).

Children who had a significant count of salivary *Lactobacillus* spp. were 4 times more likely to have dental caries than those who had not (AOR = 4.01; 95% CI = 2.24–7.18). Similarly, children with acidic saliva pH were 2 times more prone to dental caries than children have a neutral saliva pH (AOR = 1.93; 95% CI = 1.07–3.48).

Children with sweet food consumption habit were 4 times more likely to develop dental caries than who had not (AOR =4.08; 95% CI = 1.62–10.25). In parallel, those who did not clean their teeth daily were 2 times more likely to develop dental caries compared to those who had a cleaning habit. Moreover, children who did not use toothpaste in cleaning their teeth were 1.3 times more risk to develop caries than children who used toothpaste. Also, those children who had experienced dental ache were 3 times more likely to have caries than those who had not (Table 3).

## Discussion

In Ethiopia, there is a scarcity of data on dental caries amongst primary school children. Similarly, there is a lack of information on dental caries in Harar. This study revealed the prevalence of 36.9% dental caries amongst primary school children and found to be a common chronic health problem in Harar. The prevalence of dental caries found in the present study was higher than the prevalence in Bahir Dar (21.8%) [1]. This study also similar to the caries prevalence reported from Gondar (36.3%) [8]. However, it was lower than a study done in Addis Ababa (74%) [22] and Finote Selam (48.5%) [23]. In addition, our finding was lower than the studies conducted in Eritrea (78%) [24], India (59%) [25], and Qatar (85%) [26].

This study revealed the mean DMFT (0.95) was much lower than the study done in Eritrea (DMFT = 2.5) [24], Qatar (DMFT = 4.62) [26] and India (DMFT = 1.91) [25]. This DMFT value was also lower relative to the mean DMFT value stated by world oral health standards between the years of 2012–2013 [5]. To the contrary, the value was higher compared to the study done in Nigeria (DMFT = 0.45) [27] and Sudan (DMFT = 0.42) [28]. From the comparisons of above differences might be due to the diversity in dietary habits and oral hygiene practices among stated country’s communities and it might also be associated with various socio-economic and biologic risk factors [28]. The present study showed similarities to the study reported in Qatar [26] and Kenya [29] for the decay component as the primary contributor for the mean DMFT score.

Majority of the children with dental caries were able to score less (< 50%) in their previous year’s cumulative score (AOR = 3.68; 95% CI: 1.10–12.29; *p* = 0.034). This may be due to the impact of caries and associated infection that causes pain and discomfort leading to loss of attention and absence from school [9] which directly related to children’s productivity [30, 31].

Children’s grade level had a statistically significant association with dental caries. The 1st cycle (grades 1–4) students found to have 1.7 times more risk than the 2nd cycle (grades 5–8) students for dental caries. This study showed an increase in grade-level decreases the chance of dental caries. This finding was also consistent with the report from Bahir Dar showing that two times higher prevalence of dental caries amongst 1st cycle students [1]. The agreement with these findings might be because of better exposure to toothpaste by 2nd cycle students.

Sweet food consumption has significantly associated with dental caries (AOR = 4.08; 95% CI: 1.62–10.25; *p* = 0.003). This finding was in agreement with the studies reported on the sugar intake as one of the predominant predictors of caries in Addis Ababa and Finote Selam [22, 23]. The results agreement might be due to subsequent involvement of cariogenic bacteria produces copious acid by fermenting sugar in the sweet foods and leads to increased exposure of the enamel to decay [32, 33]. In consistent with the above statement, the present study revealed that *Lactobacillus* spp. count (≥ 10^5^ CFU/mL) significantly associated with dental caries (AOR = 4.01; 95% CI: 2.24–7.18; *p* = < 0.001). This finding relates to a study from Thailand (*p* < 0.001) [34] which reported high bacterial count as an indicator of high caries risk. Our finding contradicts with the study conducted in Romania (*p* = 0.131) [35] and the difference was due to different staple food and tooth cleaning habits.

Poor teeth cleaning habit was significantly associated with dental caries (AOR = 2.23; 95% CI: 1.32–3.78; *p* = 0.002). The prevalence of dental caries was higher amongst the study participants who did not have daily teeth cleaning habits (48%). It is a common fact that cleaning teeth removes away the food debris from the mouth. Therefore, *Lactobacillus* spp. and other cariogenic bacteria cannot get enough nutrients and time for growth [23, 32]. The present study reports that 40.9% of children cleaned their teeth using a traditional small wood stick (locally called mefakiya) for maintaining oral hygiene. Despite the type and nature of the traditional wood stick used in the study area, using these wood stick for teeth cleaning purposes could be the reason for the lower prevalence in the area relative to those areas with high prevalence [36].

Children who did not use the toothpaste were significantly associated with caries status in this study (AOR = 3.05; 95% CI: 1.69–5.49; *p* = 0.001). The prevalence of dental caries was lower amongst those who cleaned their teeth using brush and toothpaste (32.9%) than those who did not (39.4%). The closeness between the above proportions was may be due to lack of knowledge on how to use a toothbrush amongst the users, which were consistent with the study reported in Addis Ababa which shows 52% of children make bleed of their gum while brushing [22]. Although fluoride toothpaste is commercially available in the study area, the knowledge and the utilization of fluoride might have its own implication on the caries burden amongst toothpaste users and not users.

Dental ache is one of the common indicators of tooth decay [37]. In this study, children who had toothache were three times more likely to have dental caries (AOR = 2.95; 95% CI: 1.78–4.89; *p* = < 0.001). This finding was similar to the study conducted in Addis Ababa [22] and Sudan [28]. This might be due to the presence of a cavity before the history of pain [23]. Also, dental ache was stated as a prior reason to meet dentists [24].

### Limitations

An important limitation of this study is that no causal inferences are allowed, since we used a cross-sectional study design. Besides, the comparatively small sample size for school children population limits generalizability of the findings.

## Conclusion

The presence of significant salivary *Lactobacillus* spp. count (≥ 10^5^ CFU/mL) associated with poor academic performance, being primary cycle students, sweet diet, poor teeth cleaning habit, dental ache history, and salivary pH were the determinant factors for the burden of dental caries.

Thus, dissemination of oral hygiene awarness and access to dental care services are assumed as supreme importance for the prevention of dental caries amongst primary school children in the study area.

## Data Availability

All necessary data supporting our findings can be found in the Haramaya University repository.

## Abbreviations

AOR: Adjusted odds ratio
ATCC: American Type Culture Collection
CFU: Colony forming unit
CI: Confidence intervals
COR: Crude odds ratio
DMFT: Decayed, missing, filled tooth number
EPHI: Ethiopia Public Health Institution
IHRERC: Institutional Health Research Ethics Review Committee
MRS: Man Rogosa and Sharpe
OR: Odds ratios
SPSS: Statistical package for social science soft
WHO: World Health Organization
